# Efficacy and Safety of the Hexanic Extract of *Serenoa repens* vs. Watchful Waiting in Men with Moderate to Severe LUTS-BPH: Results of a Paired Matched Clinical Study

**DOI:** 10.3390/jcm11040967

**Published:** 2022-02-12

**Authors:** Antonio Alcaraz, Mauro Gacci, Vincenzo Ficarra, José Medina-Polo, Andrea Salonia, Jesús M. Fernández-Gómez, Alexandru Ciudin, David Castro-Díaz, Alfredo Rodríguez-Antolín, Joaquín Carballido-Rodríguez, José M. Cózar-Olmo, Santiago Búcar-Terrades, Noemí Pérez-León, Francisco J. Brenes-Bermúdez, José M. Molero-García, Antonio Fernández-Pro Ledesma, Michael Herdman, José Manasanch, Javier C. Angulo, on behalf of the QUALIPROST Study Group

**Affiliations:** 1 Urology Department, Hospital Clínic, Universitat de Barcelona, IDIBAPS, 08036 Barcelona, Spain; aalcaraz@clinic.cat; 2 Unit of Minimally Invasive and Robotic Urologic Surgery and Kidney Transplantation, Careggi University Hospital (AOUC), University of Florence, 50134 Florence, Italy; maurogacci@yahoo.it; 3 Department of Experimental and Clinical Medicine, University of Florence, 50134 Florence, Italy; 4 Department of Human and Pediatric Pathology “Gaetano Barresi”, Urology Section, University of Messina, 98125 Messina, Italy; vficarra@unime.it; 5 Urology Department, Research Institute Hospital 12 de Octubre (i+12), 28041 Madrid, Spain; jose.medina@rocurologia.com (J.M.-P.); arantolin@yahoo.es (A.R.-A.); 6 Urology Unit, HM Hospital, 28050 Madrid, Spain; 7 ROC Clinic, 28010 Madrid, Spain; 8 Division of Experimental Oncology, URI, IRCCS Ospedale San Raffaele, University Vita-Salute San Raffaele, 20132 Milan, Italy; salonia.andrea@hsr.it; 9 Unit of Urology, URI, IRCCS Ospedale San Raffaele, University Vita-Salute San Raffaele, 20132 Milan, Italy; 10 Urology Department, Hospital Universitario Central de Asturias, 33011 Oviedo, Spain; jmfergomez@gmail.com; 11 Urology Department, Hospital de Mollet, 08100 Mollet, Spain; alexciudin@gmail.com; 12 Urology Department, Hospital Universitario de Canarias, 38320 Tenerife, Spain; davidmanuelcastrodiaz@gmail.com; 13 Urology Department, Hospital Universitario Puerta de Hierro Majadahonda, 28222 Majadahonda, Spain; carballidojoaquin@gmail.com; 14 Urology Department, Hospital Universitario Virgen de las Nieves, 18014 Granada, Spain; cozarjm@yahoo.es; 15 Urology Department, Hospital El Pilar, Quirón Salud, 08006 Barcelona, Spain; sbucarterrades@gmail.com; 16 Gran Sol Primary Care Center, 08914 Badalona, Spain; noemi612@gmail.com; 17 SEMERGEN Nefro-Urology Working Group, 08338 Premià de Dalt, Spain; fjbrenesb@gmail.com; 18 San Andrés Primary Care Center, 28021 Madrid, Spain; jmolerog@gmail.com; 19 Menasalbas Primary Care Center, 45128 Toledo, Spain; afernandezprol@semg.es; 20 Insight Consulting and Research, 08301 Mataró, Spain; michael.herdman@insightcr.com; 21 Saw Swee Hock School of Public Health, National University of Singapore, Singapore 117549, Singapore; 22 Pierre Fabre Ibérica S.A., 08005 Barcelona, Spain; jmanasanch@yahoo.es; 23 Clinical Department, Universidad Europea de Madrid, 28905 Getafe, Spain; 24 Urology Department, Hospital Universitario de Getafe, 28905 Getafe, Spain

**Keywords:** moderate-severe LUTS, BPH, hexanic extract of *Serenoa repens*, watchful waiting, quality of life, sexual function, tolerability, adverse effects

## Abstract

We investigated changes in symptoms and quality of life (QoL) in men with moderate-to-severe lower urinary tract symptoms associated with benign prostatic hyperplasia (LUTS/BPH) receiving the hexanic extract of *Serenoa* *repens* (HESr) and compared results with a matched group on watchful waiting (WW). Data was from a real-world, open-label, prospective, multicenter study. This sub-group analysis included patients with moderate-to-severe symptoms receiving either the HESr 320 mg/daily for six months (HESr) or who remained untreated for LUTS/BPH (WW). Changes in urinary symptoms and QoL were measured by IPSS and BII questionnaires. Two statistical approaches (iterative matching and propensity score pairing) were used to maximize between-group comparability at baseline. Tolerability was assessed in the HESr group. After iterative matching, data for analysis was available for 783 patients (102 WW, 681 HESr). IPSS scores improved by a mean (SD) of 3.8 (4.4) points in the HESr group and by 2.2 (4.5) points in the WW group (*p* = 0.002). Changes in BII score were 1.8 (2.4) points and 1.0 (2.2) points, respectively (*p* < 0.001). Three patients (0.9%) treated with the HESr reported mild adverse effects. Moderate-severe LUTS/BPH patients treated for six months with the HESr showed greater improvements in symptoms and QoL than matched patients on WW, with a very low rate of adverse effects.

## 1. Introduction

Lower urinary tract symptoms associated with benign prostatic hyperplasia (LUTS/BPH) are very common in men aged >40 years [1,2]. The presence of such symptoms negatively impacts patients’ quality of life [3,4], as well as the lives of patients’ partners, who have been shown to suffer sleep problems and increased anxiety deriving from their partners’ LUTS, among other effects [2,5].

Several medical treatments are available to manage patients with LUTS/BPH. Alpha-adrenergic blockers (AB), 5-alpha-reductase inhibitors (5ARI), 5-phosphodiesterase inhibitors, antimuscarinic drugs, beta-3 agonists are used to relieve LUTS, though several of them can also negatively affect sexual function and/or are associated with dizziness [1,6]. Phytotherapy compounds, which form a heterogeneous group that includes extracts from plants such as *Serenoa repens*, *Pygeum africanum*, *Urtica dioica*, *Cucurbita pepo* and others, are also commonly used to treat LUTS/BPH [1]. The hexanic extract of *S. repens* (HESr) in particular has been shown to be as effective as AB [7,8,9,10,11] and 6-month treatment with 5ARI [8,9] in improving symptoms and QOL in men with LUTS/BPH, but without their negative side effects, as recently recognized in the EAU Guidelines. It is frequently administered to patients with mild or moderate LUTS/BPH who wish to avoid adverse events which may be associated with other medical treatments, especially those related to sexual function [1].

Watchful waiting (WW) is an acceptable management approach for men with mild-to-moderate non-bothersome LUTS as only a relatively small proportion progress to AUR or show complications [1]. In patients with moderate-severe LUTS/BPH included in the placebo arm of the MTOPS study [12], the cumulative incidence of overall clinical progression was reported as being 17% over 4 years. Symptom worsening was the main progression event, with a cumulative incidence of 14% over that period [13].

Clearly, one advantage of WW is that it avoids the side effects associated with treatments such as AB and 5ARIs, or interactions with drugs used to treat concurrent diseases. This is important, given that it has been shown that men undergoing medical treatment for LUTS/BPH consistently prefer treatment options with a low risk of adverse events; a recent study reported that 93% prefer a treatment with no sexual side effects [14]. In that regard, the HESr has been shown to improve symptoms and QoL in BPH patients with minimal side effects which do not impact sexual function [1,7,8,15].

Given that some patients who visit the urologist with bothersome symptoms decide to remain on WW, possibly due to concerns about the adverse effects of treatments [14], or potential drug interactions, it is of interest to evaluate how the HESr compares to WW in terms of change in symptoms and QoL, and whether additional benefits are associated with any impact on sexual function or severe AEs. To date, there are no published studies reporting a comparison of the HESr vs. WW, with adequate follow-up specifically focusing on QoL.

The objective of the present study was therefore to investigate whether 6 months of treatment with the HESr in men with moderate-or-severe LUTS/BPH can improve their QoL and symptoms without negatively affecting sexual function, and to compare the outcomes with those of a matched group on WW.

## 2. Materials and Methods

### 2.1. Patients and Study Design

Data for this analysis was from the QUALIPROST study [16] (ISRCTN11815680), a longitudinal, prospective, non-interventional, multicenter, 6-month follow-up study to evaluate change in symptoms and QoL in patients with moderate to severe LUTS/BPH (baseline IPSS score ≥8 points) managed in a urological setting. The study conformed to the Strengthening the Reporting of Observational Studies in Epidemiology (STROBE) guidelines (http://www.strobe-statement.org/; accessed on 2 October 2021) and is described in detail in Alcaraz A et al. [16]. Patients were excluded from QUALIPROST if they had received medical treatment for BPH in the 6 months prior to inclusion, if they had received any drug treatment with a known effect on BPH symptoms (i.e., diuretics, antihistamines, or tricyclic antidepressants) at any time in the 4 weeks prior to inclusion, if they had other urinary disorders, or if they had previously undergone surgery of the lower urinary tract. As QUALIPROST was a real-world study of patient management, investigators could prescribe any of the commercially available treatments according to their usual practice. Participating clinicians could also recommend WW if considered clinically appropriate, or if preferred by the patient. Informed consent was obtained individually from all patients included in the study.

Data for the present sub-analysis was from men ≥40 years with LUTS/BPH and an IPSS score ≥8 who were treated with the HESr (Permixon^®^ or Sereprostat^®^ at a recommended dose of 320 mg/day) throughout the 6-month follow-up or who remained on WW over the same period.

### 2.2. Study Variables

Key endpoints in the QUALIPROST study were change in the mean score on the International Prostate Symptom Score (IPSS) and in the impact on QoL assessed using the Benign Prostatic Hyperplasia Impact Index (BII).

The IPSS includes 8 questions, seven of which assess symptoms of LUTS/BPH, while the eighth assesses QoL associated with the condition. The symptom items assess problems with both storage (urgency, frequency, nocturia) and voiding (incomplete emptying, intermittency, weak stream, and straining to void). The overall score on the IPSS ranges from 0 to 35 for the symptom items, with a higher score indicating more severe symptoms, and from 0 to 5 for the QoL item (item 8). Separate sub-scores can also be calculated for the storage and voiding symptoms. An improvement of >3 points on IPSS items 1–7 is considered clinically relevant [17].

The BII is a self-administered questionnaire consisting of 4 questions measuring the impact of urinary symptoms on physical discomfort, worries about health, symptom bother, and interference with usual activities during the past month [18]. Items are answered using a Likert scale, with 4 or 5 response options per item and scores range from 0 (best QoL) to 13 (worst QoL). An improvement of >0.4 points on the BII score is considered clinically relevant [17].

Both the BII and the IPSS were self-completed by patients at baseline and at the 6-month follow-up visit. 

Sociodemographic data collected at baseline included age, as well as weight and height, which were used to calculate body mass index (BMI). Clinical data collected included date of onset of urinary symptoms, year of LUTS/BPH diagnosis, IPSS score, results from diagnostic tests (digital rectal exam, prostate volume, Qmax, urine analysis, serum analysis, PSA), treatment received, and information on co-morbidities and their treatment. Adverse effects potentially associated with treatment were recorded with study-specific questions at follow-up.

Treatment compliance was assessed in the HESr group using the validated Spanish version of the Haynes–Sackett questionnaire [19] which asks about: (a) patients’ difficulty taking the medication, and (b) the number of tablets they have taken in the previous month. Patients taking between 80% and 100% of the prescribed dose are considered to show good adherence.

### 2.3. Statistical Methods

To optimize comparability between the HESr and WW patients, the two groups were matched using two different methods. In the first one, an iterative matching procedure was used to ensure they were comparable at an aggregate level in terms of baseline IPSS and BII scores, maximum urinary flow (Qmax), prostate-specific antigen (PSA), and prostate volume. Patients were removed one by one from the HESr or WW group and the two groups were continually compared using *t*-tests until there were no statistically significant differences (*p* > 0.10) between them on any of the relevant baseline characteristics. To determine the success of the matching procedure, pre- and post-matching baseline between-group differences on the IPSS and BII total scores, the IPSS voiding and storage sub-scores, and IPSS item 7 (nocturia) were assessed using Student’s *t*-test for the overall sample, and for sub-groups defined by severity. The second method was a propensity score matching procedure, in which each patient in the HESr group was paired with a patient from the WW group with a similar likelihood of receiving HESr estimated using logistic regression and including the same baseline characteristics used in the iterative procedure as independent variables.

Change over time within the different treatment groups and between-group differences in the size of change on the IPSS and BII were assessed using paired *t*-tests or independent samples *t*-test, respectively. Outcomes on the IPSS storage and voiding sub-scores were also analyzed and compared between groups.

With the selected sample size of 102 controls and 680 treated patients, the study had a power of 80% to detect a 6-month minus baseline IPSS difference of at least 1.35 units, assuming a type I error of 5% and a standard deviation of 4.5 units for the between-group difference in change scores.

Quality of life (BII) analyses were performed for the samples as a whole and for two sub-groups defined by baseline severity, i.e., a moderate group with a baseline IPSS score of 8–13 and a more severe group with a baseline IPSS score of ≥14 points.

Adverse events in the HESr group were analyzed in terms of frequencies and proportions.

In all comparisons, results were considered statistically significant at *p* < 0.05. Statistical analyses were carried out using R 4.1.1 statistical software (https://www.r-project.org; accessed on 2 October 2021).

## 3. Results

### 3.1. Iterative Matching Sample

**Figure 1:**
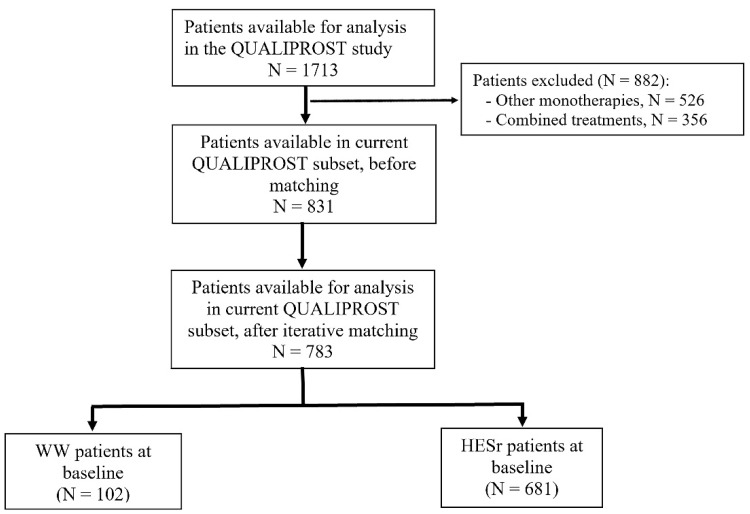
Study flow-chart (iterative matching sample). HESr: hexanic extract of *Serenoa repens*; WW: watchful waiting.

After iterative matching, data was available for analysis from a total of 783 patients (681 HESr, 102 WW) (Figure 1). Table 1 shows the sociodemographic and clinical characteristics for the HESr and WW groups at baseline. After the matching process, there were no statistically significant differences between the two groups on any of the parameters analyzed. In the HESr group, 37.7% of the patients (*n* = 224) at baseline had a prostate volume > 40 cm^3^ and a PSA ≥ 1.5 ng/mL compared to 43.5% (*n* = 40) in the WW group (*p* = 0.346) (Table S1) showing the percentage of patients with risk of progression in each group. The percentage of patients with severe LUTS/BPH was 14.7% and 17.2% in the WW and HESr groups, respectively. Rates of concomitant diseases at baseline were similar in the groups, with hypertension and dyslipidemia being the most prevalent (Table S2).

**Figure 2:**
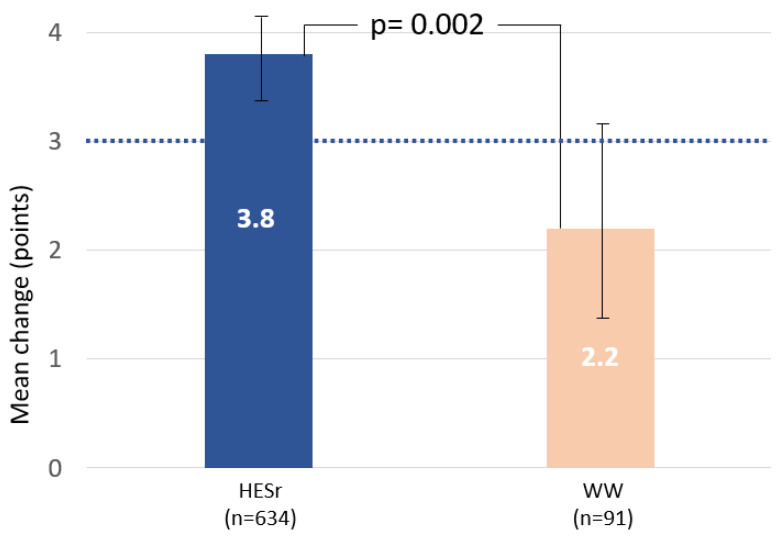
Mean (95% CI) improvement in IPSS total score from baseline to 6 months for the HESr and WW groups. Dotted line indicates the level at which a clinically relevant difference is observed. HESr: hexanic extract of *Serenoa repens;* WW: watchful waiting; IPSS: International Prostate Symptom Score.

Table 2 shows the change in symptoms and QoL for the HESr and WW groups after six months of follow-up. The IPSS improvement was larger in the HESr group with a mean (SD) improvement of 3.8 (4.4) compared to 2.2 (4.5) in the WW group (*p* = 0.002). The changes correspond to a mean percentage improvement on the IPSS of 25% in the HESr group versus a change of 15.3% in the WW group. Symptom relief on the IPSS voiding and storage sub-scores were also showed a statistically significant improvement greater in the HESr group compared to patients in WW (*p* < 0.05). Mean change on the IPSS together with 95% CI is shown in Figure 2 for the two study groups.

**Figure 3:**
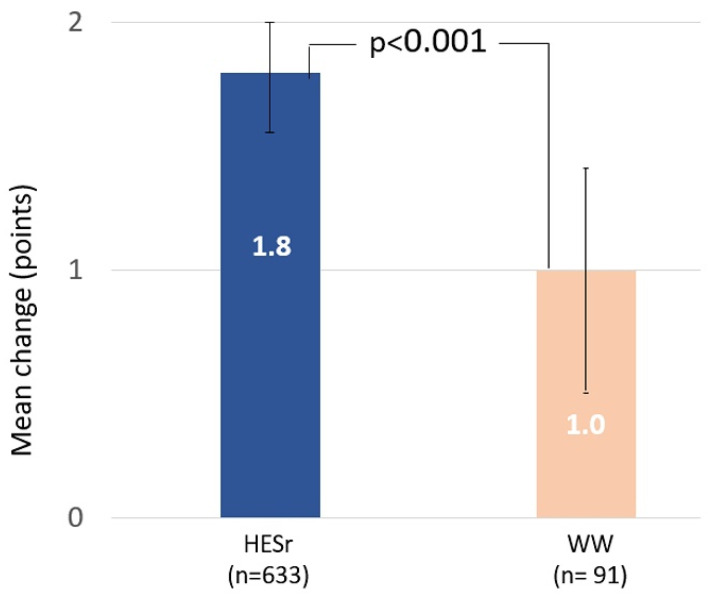
Mean (95% CI) improvement in BII total score from baseline to 6 months for the HESr and WW groups. HESr: hexanic extract of *Serenoa repens*; WW: watchful waiting; BII: Benign Prostatic Hyperplasia Impact Index.

The improvement in QoL was also greater in the HESr group, with a total mean (SD) change score of 1.8 (2.4) points on the BII, representing a 30% improvement, compared to a mean change of 1.0 (2.2) point, or a 17.5% improvement, in the WW group (*p* < 0.001). BII change for each study group with its 95% CI is shown in Figure 3. The HESr group showed statistically significant greater improvements on 3 of the 4 BII items compared to those on WW, with larger reductions in score (improved QoL) on the items assessing concern about health, bother with urination, and impact on daily activities (Table 2). When analyzing the BII results based on the severity of baseline symptoms (IPSS 8–13 or IPSS ≥ 14), we observed that the improvement in QoL was greater in the HESr patients than in the WW group in both cases (*p* < 0.04, Table S3).

As this was an observational study in which clinicians applied their usual criteria for requesting clinical tests, the number of patients with Qmax and PSA data available at follow-up was substantially smaller than the number with full IPSS and BII results at the same time point. In the WW group, the number of patients with follow-up Qmax and PSA data (*n* = 20) was too small to allow for appropriate analysis, so follow-up results are not provided here. Good adherence was reported by 98.7% of the patients in the HESr group. 

Six (0.9%) patients reported AEs, with five patients (0.7%) reporting constipation and one patient (0.2%) nausea.

### 3.2. Propensity Score Matching

The propensity score sample was composed of a total of 140 patients (92 HESr, 48 WW) (Figure S1). There were no statistically significant differences in any baseline characteristics between the two study groups (Table S4) nor in the percentage of patients showing risk of progression characteristics in each group (Table S5). Prevalence of concomitant diseases at baseline is described in Table S6.

Results from the propensity score-matched sample were very similar to those reported for the iteratively matched sample and are shown in Table S7. IPSS (SD) scores improved by 4.2 (4.2) points in the HESr group and by 1.6 (3.3) points in the WW patients (*p* < 0.001) (Figure S2). IPSS voiding and storage sub-scores also improved significantly in the HESr group compared to the WW group (*p* < 0.004). For QoL, mean (SD) BII scores improved by 2.1 (2.3) and 0.8 (1.7) points in the HESr and WW groups, respectively (*p* < 0.001) (Figure S3). The biggest differences between the two study groups were on the BII items assessing bother with urination, and limitations on daily activities.

The pattern of AEs reported was similar to that seen in the iteratively matched sample, with 3 patients (3.3%) reporting an AE, either constipation (2 patients, 2.2%) or nausea (1 patient, 1.1%).

## 4. Discussion

To date, this is the first study to compare changes in symptoms and QoL in patients treated with the HESr vs. WW. Patients included had moderate-severe LUTS/BPH at baseline and data were collected in a real-world setting, which may make the results more generalizable. After six months of follow-up, patients receiving the HESr reported better outcomes in terms of symptoms and QoL than patients managed with watchful waiting. The focus on QoL in the present study and the inclusion of patients with moderate-severe symptoms adds to evidence from earlier studies which studied the effects of the HESr in patients with mild symptoms [20], or reported the effect on clinical symptoms, rather than QoL [21,22], or which focused on the HESr’s anti-inflammatory action [23].

The improvement in symptoms observed in the present sub-analysis was similar to that previously observed in RCTs which have investigated the HESr. In particular, Debruyne et al. [7] reported a decrease in IPSS score of 4.4 points in patients receiving the HESr after 12 months of treatment and Latil et al. [24] found a reduction of 4.3 points on the IPSS in patients receiving the HESr. The reduction in IPSS score is in line with that seen in previous articles evaluating the efficacy of the HESr. In a study comparing the HESr with tamsulosin to treat LUTS/BPH, the IPSS score improved by 4.5 points in the HESr group [11]. In another publication comparing the HESr and tamsulosin versus a combination of the two, the IPSS score decreased by 5.4 points in the HESr group [10].

Other studies have reported no difference between an alcoholic extract of *S. repens* [25] or carbon dioxide *Serenoa repens* extract [26] and placebo in terms of the effect on symptoms and placebo. However, it should be noted that, as stated in the EAU Guidelines [1], there are differences between these extracts and the HESr which means that findings observed with one brand of *S. repens* cannot be extrapolated to other brands. In a recently updated report, the European Medicines Agency states that, as opposed to alcoholic and carbon dioxide extracts, the HESr is the sole *S. repens* extract with sufficient evidence to support it as well-established for treatment of LUTS/BPH [15]. 

Another important finding of the present analysis is that patients receiving the HESr showed greater improvement in QoL than those on WW. A decrease of >0.4 points on the BII has been shown to be considered relevant by patients [17], whereas the difference in the size of change on the BII between our two study groups was 0.8 points, which considerably exceeds that threshold, in favor of the HESr group. The improvement of 1.8 points on the BII in the HESr group is similar to that observed for other drugs used to treat LUTS/BPH, such as dutasteride and tamsulosin, which showed improvements of 1.7 points and 1.5 points, respectively, after 2 years of treatment in the COMBAT trial [27]. Improvements on the BII were reported in other publications evaluating the efficacy of the HESr; Alcaraz et al. [11] reported a mean reduction of 2.2 points for patients receiving the HESr in a comparison with tamsulosin and a mean improvement of 2.7 points in an analysis comparing the HESr with tamsulosin and with a combination of both drugs [10].

A question that arises when analyzing these data is why patients with moderate-or-severe LUTS who visit a urologist, and who have a risk of progression (i.e., prostate volume > 40 cm^3^ and a PSA ≥ 1.5 ng/mL) in over 43% of cases, do not receive medical treatment. In the case of the QUALIPROST Study, such patients represented 8.9% of the total sample [16]. Reasons may include a fear of suffering adverse effects, especially on sexual function, or a desire to avoid the risk of interactions with other drugs the patient is taking; approximately 40% of patients in the QUALIPROST sample had at least one concomitant disease. A recent publication [14] indicated that men with LUTS prefer to receive a drug treatment that presents a low risk of erectile dysfunction or adverse effects related to sexual function. Other studies have reported that approximately 20% of LUTS/BPH patients treated for 12 months abandon treatment due to adverse events [28]. If clinicians and patients are aware of a medication, the HESr which is recommended in the EAU Guidelines as a treatment “for men with LUTS who want to avoid any potential adverse events especially related to sexual function” [1] and which is associated with symptom relief and improved QoL without any known drug interactions, that could potentially facilitate treatment adherence.

Finally, it should be borne in mind that LUTS affects not only patient QoL but also that of their partners. For example, Mitropoulos and colleagues found that sleep impairment was reported by 28% of partners of patients with symptomatic BPH, and alteration of social activities by 30% [5]. Findings were correlated with IPSS scores, and sex life can also be affected. Therefore, a spillover effect of the improvement seen in patients’ LUTS could be an improvement in their partners’ QoL, though that requires further investigation.

As has been shown in several previous studies [7,10,11,16], the improvements in symptoms and QoL associated with the HESr do not come at the expense of any relevant incidence of adverse effects. In the present sub-analysis, only 1% of patients receiving HESr reported an AE, and they were all classified as mild. This is an important point to consider when discussing treatment options with patients.

Strengths of the present study include the matching approaches used, which, as indicated by the lack of any statistically significant differences between groups at baseline, ensured a high level of comparability between the treatment arms. The fact that two different types of matching approaches showed similar outcomes also helps to support the reliability of the results. Another strength is that the analysis used data collected in conditions of daily clinical practice, which means the findings may better reflect patient experience than findings from randomized clinical trials, which are often performed under very controlled conditions. 

The present study had also some limitations. Data were obtained without the use of randomization or blinding; patients were therefore allocated to a specific management approach based on clinician judgment and shared decision-making with patients. This could lead to a selection bias although the use of two matching strategies should help to minimize any potential bias. The limited percentage of uroflowmetries, which is similar for both groups, could also be considered a limitation although it reflects current clinical practice, which was one of the objectives of the study. Finally, another limitation could be the unbalanced baseline sample as there were more patients available in the HESr group than in the WW one, using the iterative matching approach. However, selecting a larger sample in this approach also increased statistical power. Furthermore, the propensity score matching procedure led to a more balanced sample in terms of numbers in the two groups and the results are similar to those observed using the iterative matching procedure, with no relevant differences.

Carrying out an RCT in men with moderate/severe LUTS/BPH who refuse medical treatment for their condition to avoid potential adverse events associated with LUTS/BPH drugs or drug interactions would be of interest.

## 5. Conclusions

As an initial treatment option in men with moderate to severe LUTS/BPH, the HESr shows relevant advantages over WW as it is associated with clinically significant improvements in both urinary symptoms and QoL with a negligible rate of only mild adverse effects.

## Figures and Tables

**Table 1:** Patient baseline characteristics by study group (iterative matching sample).

	HESr		WW		*p* Value
	N	Mean (SD)	N	Men (SD)	
Age, years	610	61.7 (9.1)	74	61.9 (9.4)	0.868
BMI, Kg/m^2^	604	26.5 (2.9)	72	26.3 (2.5)	0.647
IPSS	681	15.2 (5.0)	102	14.4 (4.5)	0.114
*IPSS voiding subscore*	681	8.6 (3.2)	102	8.2 (3.1)	0.208
*IPSS storage subscore*	681	6.6 (2.4)	102	6.2 (2.0)	0.117
BII	681	6.0 (2.6)	102	5.7 (2.5)	0.237
Time since diagnosis, years	608	1.1 (2.6)	74	1.4 (2.7)	0.402
Qmx, mL/s	338	13.9 (3.9)	50	13.7 (3.6)	0.701
Prostate volume, cc	584	43.3 (15.8)	91	46.2 (15.9)	0.107
PSA, ng/mL	630	2.1 (1.3)	98	2.1 (1.3)	0.840

HESr: hexanic extract of *Serenoa repens*; WW: watchful waiting; BMI: body mass index; IPSS: International Prostate Symptom Score; BII: Benign Prostatic Hyperplasia Impact Index; Qmax: maximum urinary flow rate; PSA: prostate-specific antigen.

**Table 2:** Improvements from baseline to 6-month follow-up in symptoms and quality of life (iterative matching sample).

		HESr			WW		*p* Value
	N	Mean (SD)	% *	N	Mean (SD)	% *	
IPSS total	634	3.8 (4.4)	25.0	91	2.2 (4.5)	15.3	0.002
*IPSS voiding subscore*	634	2.1 (2.9)	24.4	91	1.0 (2.8)	12.2	0.001
*IPSS storage subscore*	634	1.7 (2.1)	25.8	91	1.2 (2.1)	19.4	0.039
BII total	632	1.8 (2.4)	30.0	91	1.0 (2.2)	17.5	<0.001
*Physical discomfort*	631	0.4 (0.8)	26.7	91	0.3 (0.8)	20.0	0.119
*Worry about the health*	631	0.5 (0.9)	31.3	91	0.3 (0.7)	18.8	0.002
*Bothered with urination*	631	0.4 (0.8)	28.6	91	0.2 (0.7)	15.4	0.015
*Daily activity limitation*	631	0.5 (0.8)	35.7	91	0.2 (0.8)	15.4	0.002

HESr: hexanic extract of *Serenoa repens*; WW: watchful waiting; IPSS: International Prostate Symptom Score; BII: Benign Prostatic Hyperplasia Impact Index; * Percentage of improvement over initial values.

## Data Availability

Full data will be provided by correspondence author upon reasonable request.

## Supplementary Materials

The following supporting information can be downloaded at: https://www.mdpi.com/article/10.3390/jcm11040967/s1. Figure S1: Study flow-chart (propensity score matching); Figure S2: Mean (95% CI) improvement in IPSS total score from baseline to 6 months for the HESr and WW groups (propensity score sample); Figure S3: Mean (95% CI) improvement in BII total score from baseline to 6 months for the HESr and WW groups (propensity score sample); Table S1: Patients with prostate volume > 40 cm^3^ and/or PSA ≥ 1.5 ng/mL at baseline by treatment group, in the iterative matched sample; Table S2: Concomitant diseases at baseline by treatment group (iterative matching sample); Table S3: Quality of life improvement by means of BII according to IPSS baseline value by treatment group (iterative matching sample); Table S4: Patient baseline characteristics by study group (propensity score sample); Table S5: Patients with prostate volume >40 cm^3^ and/or PSA ≥1.5 ng/mL at baseline by treatment group (propensity score sample). Table S6: Concomitant diseases at baseline by treatment group (propensity score sample); Table S7: Improvements from baseline to 6-month follow-up in symptoms and quality of life (propensity score sample).

## Appendix A

The following investigators participated in the QUALIPROST Study Group:

A Coruña: Dr. L. Busto Castañón; Dr. J.E. Duarte Novo; Dr. M. Montes Couceiro; Dr. A. Rodríguez Alonso; Alicante: Dr. J.A. Canovas Ivorra; Dr. C. López López; Dr. L. Prieto Chaparro; Almería: Dr. F. Gómez Berjón; Dr. J. Hortelano Parras; Asturias: Dr. A. M. Huescar; Dr. P.L. Muntañola Armora; Badajoz: Dr. E. Godoy Rubio; Dr. J. Mariño del Real; Barcelona: Dr. J. Armona Mani; Dr. F.J. Blasco Casares; Dr. Ll. Cortadellas Angel; Dr. J. Fernández Zuazu; Dr. J.M. Malet Carreras; Dr. S. Mando Dakkar; Dr. J.M. Prats Puig; Dr. M. Puyol Pallás; Dr. J.A. Romero Martín; Dr. J. Sáenz de Cabezón Martí; Dr. J. Sánchez Macías; Dr. C. Vargas Blasco; Dr. J.M. Vayreda Martija; Bilbao: Dr. J.A. Gallego Sánchez; Dr. N. Prieto Ugidos; Cádiz: Dr. J.M. Arroyo Maestre; Dr. S. Garrido Insua; Dr. A. Gutiérrez de Pablo; Dr. M. Marmol Ruíz; Dr. F. Reyes Martínez; Castellón: Dr. J. Beltrán Persiva; Dr. L. Monzonis Rey; Dr. J. Palacios Castañeda; Ciudad Real: Dr. N. Jiménez López-Lucendo; Dr. D. Rodríguez Leal; Córdoba: Dr. J.C. Regueiro López; Granada: Dr. M. Cabezas Zamora; Guipúzcoa: Dr. G. Garmendia Olaizola; Dr. J.A. Rodríguez Andrés; Jaén: Dr. J. Jiménez Verdejo; Dr. E.J. Zarate Rodríguez; León: Dr. S.C. Gómez Cisneros; Dr. M. Lozano Rebollo; Lleida: Dr. J. Cortada Robert; Dr. L.M. Flavian Domenech; Logroño: Dr. A. Fernández Fernández; Lugo: Dr. F.J. Neira Pampin; Madrid: Dr. A. Abdallah Merhi; Dr. F. Arias Funes; Dr. I.T. Castillón Vela; Dr. J.M. Duarte Ojeda; Dr. M.J. García-Matres Cortés; Dr. J.F. Hermina Gutierrez; Dr. J.J. López-Tello García; Dr. C. Martín García; Dr. B. M.El-Awadeh; Dr. J.D. Rendón Sánchez; Dr. R. Rodríguez-Patron Rodríguez; Dr. J.C. Ruíz de la Roja; Dr. J. Vallejo Herrador; Dr. D. Vázquez Alba; Málaga: Dr. A. Bonilla Maldonado; Dr. J.M. Fernández Montero; Dr. A. Galacho Bech; Dr. C. Marchal Escalona; Dr. P. Rodero García; Dr. G. Sanz; Dr. J.D. Vázquez Cervilla; Murcia: Dr. G. Server Pastor; Pontevedra: Dr. D. Jamardo González; Dr. A. Selas Pérez; Salamanca: Dr. F. Díaz Alférez; Dr. M.F. Lorenzo Gómez; Santander: Dr. J.A. Portillo Martín; Sevilla: Dr. F.J. Giráldez Puig; Dr. M.A. Gutiérrez González; Dr. J.I. Huesa Martínez; Dr. Y. Ismail Tomaizeh; Dr. J. Leal Arenas; Dr. J. Martín Calero; Dr. J.L. Moyano Calvo; Dr. A. Ortiz Gámiz; Dr. J.M. Poyato Galán; Dr. J.A. Valero Puerta; Dr. E. Vilches Cocovi; Tarragona: Dr. P.L. Álvarez de la Red; Dr. J. Benagues Pamies; Dr. M. Prados Saavedra; Tenerife: Dr. T. Concepción Masip; Dr. C. Gómez de Segura Melcon; Dr. E. González de Chaves; Dr. J. Postius Robert; Toledo: Dr. F. Álvarez Fernández; Dr. A. Iglesias Justo; Dr. A. Melchor Galán; Valencia: Dr. J.E. Blasco Alfonso; Dr. M.A. Bonillo García; Dr. A. Collado Serra; Dr. J.A. García Cebrián; Dr. L.García Reboll; Dr. Y. Pallás Costa; Dr. M.J. Sánchez Sanchiz; Dr. J. Santamaría Meseguer; Zaragoza: Dr. A. García de Jalón Martínez; Dr. J.M. Gil Fabra; Dr. F. Monzón Alebesque; Dr. A. Ucar Terren.
