# Supplementary material for: Efficacy and Safety of the Hexanic Extract of Serenoa repens vs. Watchful Waiting in Men with Moderate to Severe LUTS-BPH: Results of a Paired Matched Clinical Study
Source: J Clin Med. 2022 Feb 12;11(4):967. doi: 10.3390/jcm11040967 (PMC8878824; doi:10.3390/jcm11040967)
Supplement: Supplementary file 1 [file jcm-11-00967-s001.zip › jcm-1543549-supplementary.pdf]

Supplementary Materials

Table S1. Patients with prostate volume >40 cm^3 and/or PSA ≥1.5 ng/ml at baseline by treatment group, in the iterative matched sample.

	**HESr**<br>n (%)*	**WW**<br>n (%)*	**p value**
PV >40 cm^3	299 (51.2)	50 (54.9)	0.581
PSA ≥1.5 ng/ml	413 (65.6)	63 (64.3)	0.895
PV >40 cm^3 and PSA ≥1.5 ng/ml	224 (37.7)	40 (43.5)	0.346

HESr*:* hexanic extract of *Serenoa repens*; WW: watchful waiting; PV: Prostate volume; PSA: Prostate-Specific Antigen.
* % calculated over the number of patients with a test at baseline

Table S2. Concomitant diseases at baseline by treatment group (iterative matching sample).

	**HESr**<br>(n = 681)	**WW**<br>(n = 102)	**p value**
Patients with any concomitant illness, n (%)	217 (31.9)	39 (38.2)	0.244
High blood pressure, n (%)	105 (15.4)	16 (15.7)	1.000
Dyslipidemia, n (%)	82 (12.0)	12 (11.8)	1.000
Diabetes mellitus, n (%)	52 (7.64)	14 (13.7)	0.061
Other, n (%)	15 (2.20)	0 (0.00)	0.240

HESr*:* hexanic extract of *Serenoa repens*; WW: watchful waiting.

Table S3. Quality of life improvement by means of BII according to IPSS baseline value by treatment group (iterative matching sample).

	**HESr**			**WW**			**p value**
**Initial IPSS**	**n**	**Mean (SD)**	**%***	**n**	**Mean (SD)**	**%***	
8-13	267	1.2 (1.9)	25.5	42	0.3 (1.8)	7.3	0.005
≥14	365	2.3 (2.6)	32.9	49	1.6 (2.4)	22.9	0.038

WW: watchful waiting; HESr*:* hexanic extract of *Serenoa repens;* IPSS: International Prostate Symptom Score; BII: Benign Prostatic Hyperplasia Impact Index. P value for between-group difference in change scores.
* Percentage of improvement over initial values.

Table S4. Patient baseline characteristics by study group (propensity score sample).

	HESr		WW		p value
	n	mean (SD)	n	mean (SD)	
Age, years	85	62.8 (8.2)	25	61.7 (8.0)	0.563
BMI (Kg/m^2)	84	26.5 (3.0)	24	26.7 (2.5)	0.708
IPSS	92	15.1 (4.3)	48	14.7 (4.3)	0.604
IPSS voiding subscore	92	8.8 (2.6)	48	8.4 (3.0)	0.502
IPSS storage subscore	92	6.3 (2.1)	48	6.3 (2.0)	0.879
BII	92	6.6 (2.2)	48	6.2 (2.1)	0.285
Time since diagnosis (years)	85	1.0 (2.5)	25	0.8 (237)	0.693
Qmax (ml/s)	92	13.7 (4.2)	48	13.6 (3.6)	0.911
Prostate volume (cm^3)	92	47.5 (14.9)	48	49.6 (14.9)	0.450
PSA (ng/ml)	92	2.2 (1.1)	48	2.3 (1.1)	0.471

HESr*:* hexanic extract of *Serenoa repens*; WW: watchful waiting; BMI: body mass index; IPSS: International Prostate Symptom Score; BII: Benign Prostatic Hyperplasia Impact Index; Qmax: maximum urinary flow rate; PSA: prostate-specific antigen.

Table S5. Patients with prostate volume >40 cm^3 and/or PSA ≥1.5 ng/ml at baseline by treatment group (propensity score sample).

	HESr<br>n (%)*	WW<br>n (%)*	p value
PV >40 cm^3	55 (59.8)	32 (66.7)	0.539
PSA ≥1.5 ng/ml	63 (68.5)	35 (72.9)	0.727
PV >40 cm^3 and PSA ≥1.5 ng/ml	42 (45.7)	27 (56.2)	0.311

HESr*:* hexanic extract of *Serenoa repens*; WW: watchful waiting; PV: Prostate volume; PSA: Prostate-Specific Antigen.

* % calculated over the number of patients with a test at baseline

Table S6. Concomitant diseases at baseline by treatment group (propensity score sample).

	**HESr** (n = 92)	**WW** (n = 48)	**p value**
Patients with any concomitant illness, n (%)	35 (38.0)	25 (52.1)	0.158
High blood pressure, n (%)	20 (21.7)	10 (20.8)	1.000
Dyslipidemia, n (%)	15 (16.3)	4 (8.3)	0.295
Diabetes mellitus, n (%)	10 (10.9)	14 (29.2)	0.013
Other, n (%)	2 (2.17)	0 (0.00)	0.546

HESr*:* hexanic extract of *Serenoa repens*; WW: watchful waiting.

Table S7. Improvements from baseline to 6-month follow-up in symptoms and quality of life (propensity score sample)

		**HESr**			**WW**		**p value**
	**n**	**Mean (SD)**	**%***	**n**	**Mean (SD)**	**%***	
IPSS total	85	4.2 (4.2)	27.8	44	1.6 (3.3)	10.9	<0.001
IPSS voiding sub-score	85	2.4 (2.6)	27.3	44	0.7 (2.3)	8.3	<0.001
IPSS storage sub-score	85	1.8 (2.2)	28.6	44	0.8 (1.6)	12.7	0.003
BII total	85	2.1 (2.3)	31.8	44	0.8 (1.7)	12.9	<0.001
Physical discomfort	85	0.5 (0.6)	29.4	44	0.2 (0.7)	11.7	0.016
Worry about the health	85	0.6 (0.8)	33.3	44	0.3 (0.6)	16.7	0.061
Bothered with urination	85	0.5 (0.7)	31.3	44	0.1 (0.5)	7.7	<0.001
Daily activity limitation	85	0.6 (0.9)	37.5	44	0.1 (0.7)	71.4	<0.001

HESr*:* hexanic extract of *Serenoa repens;* WW: watchful waiting; IPSS: International Prostate Symptom Score; BII: Benign Prostatic Hyperplasia Impact Index. P value for between-group difference in change scores.

* Percentage of improvement over initial values.

Figure S1. Study flow-chart (propensity score matching)

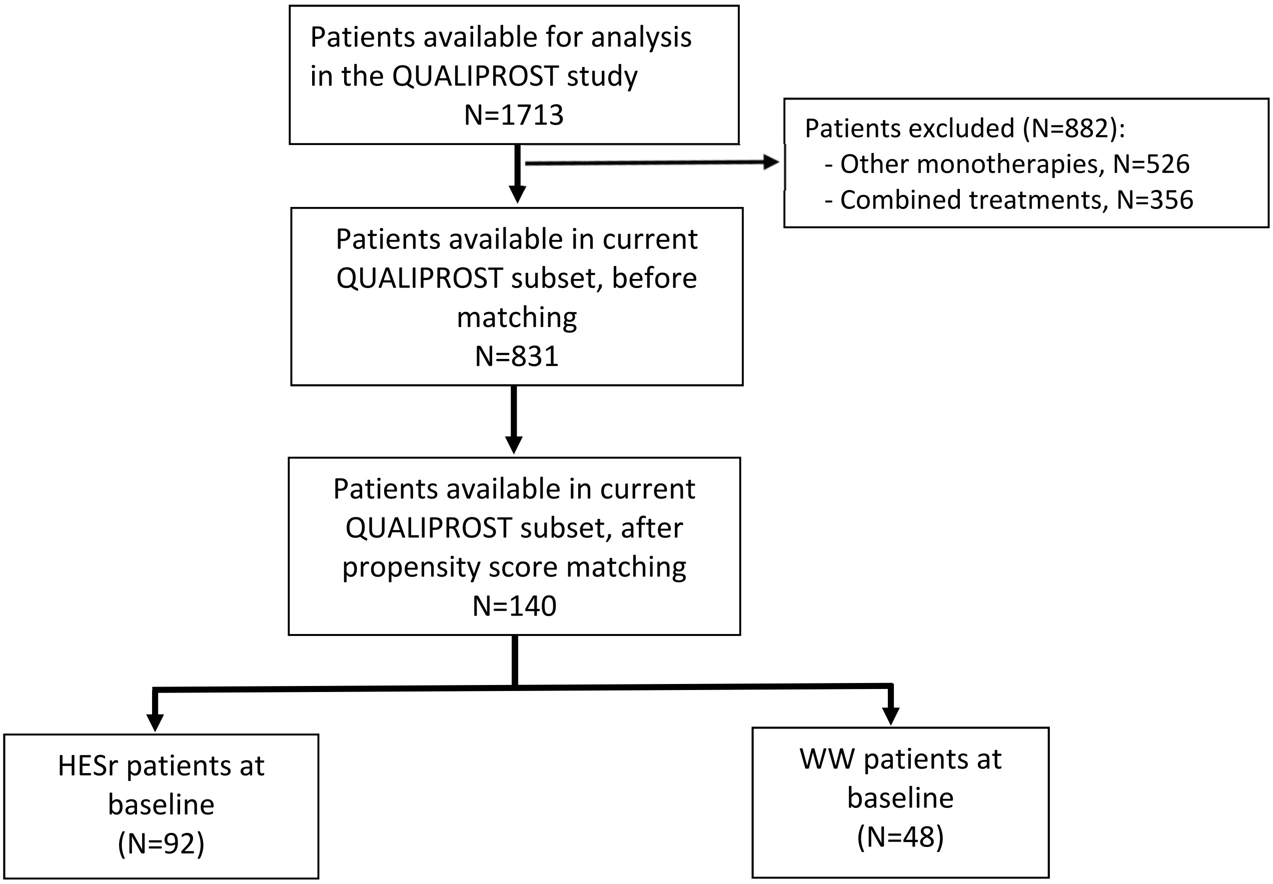

HESr: hexanic extract of *Serenoa repens*; WW: watchful waiting

Figure S2. Mean (95% CI) improvement in IPSS total score from baseline to 6 months for the HESr and WW groups (propensity score sample).

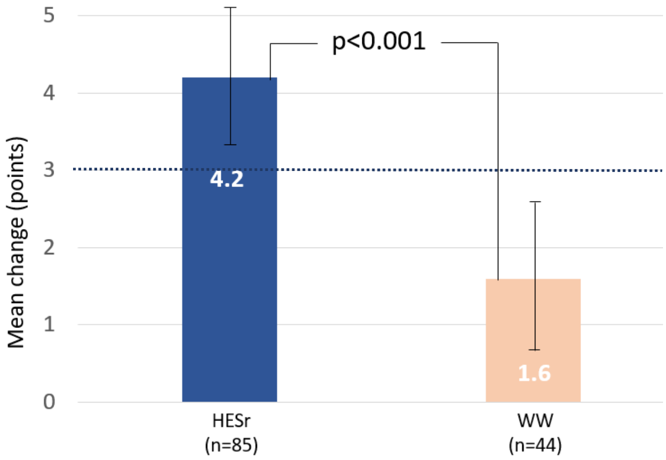

Dotted line indicates the level at which a clinically relevant difference is observed.

HESr*:* hexanic extract of *Serenoa repens;* WW: watchful waiting; IPSS: International Prostate Symptom Score.

Figure S3. Mean (95% CI) improvement in BII total score from baseline to 6 months for the HESr and WW groups (propensity score sample).

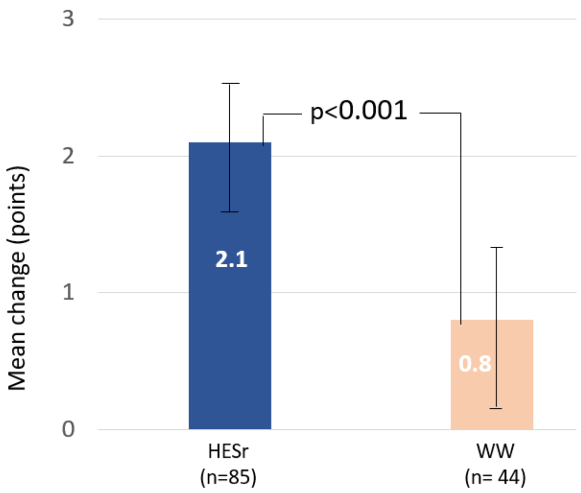

HESr*:* hexanic extract of *Serenoa repens;* WW: watchful waiting; BII: Benign Prostatic Hyperplasia Impact Index.
